# Broad patterns in domestic vector-borne *Trypanosoma cruzi* transmission dynamics: synanthropic animals and vector control

**DOI:** 10.1186/s13071-015-1146-1

**Published:** 2015-10-22

**Authors:** Jennifer K. Peterson, Sarah M. Bartsch, Bruce Y. Lee, Andrew P. Dobson

**Affiliations:** Department of Ecology and Evolutionary Biology, Princeton University, Princeton, NJ USA; Public Health Computational and Operations Research (PHICOR), John Hopkins Bloomberg School of Public Health, Baltimore, MD USA

**Keywords:** *Trypanosoma cruzi* transmission dynamics, Chagas disease, *Trypanosoma cruzi*, Triatomine bugs, Synanthropic animals, Vector control, 2020 goals, Neglected Tropical Diseases

## Abstract

**Background:**

Chagas disease (caused by *Trypanosoma cruzi)* is the most important neglected tropical disease (NTD) in Latin America, infecting an estimated 5.7 million people in the 21 countries where it is endemic. It is one of the NTDs targeted for control and elimination by the 2020 London Declaration goals, with the first goal being to interrupt intra-domiciliary vector-borne *T. cruzi* transmission. A key question in domestic *T. cruzi* transmission is the role that synanthropic animals play in *T. cruzi* transmission to humans. Here, we ask, (1) do synanthropic animals need to be targeted in Chagas disease prevention policies?, and (2) how does the presence of animals affect the efficacy of vector control?

**Methods:**

We developed a simple mathematical model to simulate domestic vector-borne *T.cruzi* transmission and to specifically examine the interaction between the presence of synanthropic animals and effects of vector control. We used the model to explore how the interactions between triatomine bugs, humans and animals impact the number and proportion of *T. cruzi-*infected bugs and humans. We then examined how *T. cruzi* dynamics change when control measures targeting vector abundance are introduced into the system.

**Results:**

We found that the presence of synanthropic animals slows the speed of *T. cruzi* transmission to humans, and increases the sensitivity of *T. cruzi* transmission dynamics to vector control measures at comparable triatomine carrying capacities. However, *T. cruzi* transmission is amplified when triatomine carrying capacity increases with the abundance of syntathoropic hosts.

**Conclusions:**

Our results suggest that in domestic *T. cruzi* transmission scenarios where no vector control measures are in place, a reduction in synanthropic animals may slow *T. cruzi* transmission to humans, but it would not completely eliminate transmission. To reach the 2020 goal of interrupting intra-domiciliary *T. cruzi* transmission, it is critical to target vector populations. Additionally, where vector control measures are in place, synanthropic animals may be beneficial.

## Background

Chagas disease (etiol. agent *Trypanosoma cruzi*)*,* is a neglected tropical disease (NTD) endemic to the Americas, where it is vector-borne by triatomine bugs, subfamily Triatominae. An estimated 5.7 million people are infected with Chagas disease in 21 Latin American countries [1], with 10,000 Chagas-related deaths per year [2]. Thirteen percent of the Latin American population are at risk of infection [1]. Globally, Chagas disease is estimated to cost $627.5 million in healthcare costs annually and result in ~806,000 DALYs, with currently-infected individuals generating $24.7 billion in healthcare costs and 29.4 million DALYs over their lifetime [3]. With this substantial burden, Chagas disease is one of the ten NTDs targeted for control or elimination by 2020. The World Health Organization (WHO) has proposed seven milestones to combat Chagas disease by 2020, the first of which is to interrupt intra-domiciliary vector-borne transmission in Latin America [4, 5] via spraying with indoor residual insecticides (IRS) and improving housing conditions. While dwelling/housing improvement is effective in reducing *T. cruzi* transmission [6], it is generally expensive and time consuming. IRS can also be effective in reducing *T. cruzi* infection prevalence in humans [7, 8], but requires repeated spraying to avoid vector re-infestation, which can be resource intensive. To meet the 2020 Chagas control goals, these methods should be applicable and achievable across the diversity of epidemiological and ecological settings of endemic Chagas disease.

One important aspect of domestic transmission that is not included in the strategies for meeting the 2020 goal of interrupting domestic vector borne *T.cruzi* is the presence of synanthropic animals. These are animals that are associated with humans, whether as pets, livestock or pests. Some of these species are viable *T. cruzi* hosts (e.g., dogs [9, 10]), but they also include non-competent species (e.g., chickens [11]) that serve as triatomine food sources. Synanthropic animals are not currently included in the strategies for meeting the 2020 goals, yet a large body of empirical and theoretical work suggests they have a significant effect on Chagas disease dynamics ([9–17]).

Here we develop a simple model to simulate domestic vector-borne *T. cruzi* transmission, and more specifically, to examine the interaction between the presence of synanthropic animals and effects of vector control. We first use the model to explore how the dynamic interactions between triatomine bugs, humans and animals impact the number and proportion of *T. cruzi-*infected bugs and humans. As the epidemiology of Chagas disease differs widely across its range with several different vector species (within the subfamily Triatominae) and mammalian reservoir species, we have deliberately simplified our model to only consider human hosts, a single species of vector, and a homogeneous pool of reservoir hosts that vary in abundance with one division between viable and non-viable hosts. Although different synanthropic host have diverse life expectancies within and between species, as well as variable levels of *T. cruzi* competence, we have assumed here that these complexities can be captured by aggregating these differences into a pool of viable and non-viable hosts. The model we describe can be readily tailored to situations where the abundances of different synanthropic host species have been quantified.

To examine the effect of current policies on domestic transmission, we add in vector control methods that target vector death rates (e.g., IRS) and carrying capacity (e.g., home improvement). We aim to answer the following questions: (1) do synanthropic animals need to be targeted in Chagas disease prevention policies?, and (2) how does the presence of animals affect the efficacy of vector control?

## Methods

### Model structure

We assumed that the essential dynamics of Chagas disease, (defined as *T. cruzi* infection in humans only), could be captured using six coupled ordinary differential equations that describe the abundance of actively feeding triatomine bugs, changes in the numbers of humans with Chagas disease, and the abundance of infected synanthropic animals (of multiple, unspecified species), a proportion of which are viable *T. cruzi* hosts. The model was run using R software version 3.03 [18]. Ordinary differential equations were solved in R using the ‘deSolve’ package [19].

### Triatomine bug dynamics

Equations 1 and 2 describe the dynamics of the triatomine bug population, which we divided into uninfected bugs, (B), and infected ‘vectors’, (V). Bugs are born at a per capita rate, *r*, with vectors having a modified birth rate, *fr*. We assume that birth rates are reduced as the total bug population approaches a finite carrying capacity, K. Vectors, V, are assumed to have fed on an infected host, and sufficient time has elapsed for the vector to become infectious at subsequent blood meals. Bugs move into the vector class through the ingestion of *T. cruzi-*infected blood meals from humans in one of three infection classes, (described in detail below), or infected animals (*I*_*R*_), at a rate of *βc*_*x*_, where *β* represents the human-triatomine contact rate, and *c*_*x*_ represents the probability of infection upon contact. The probabilities of infection are unique to each infection class, while the contact rate is the same, as we assume homogeneous mixing. Our current model ignores co-infection at subsequent blood meals, a complexity that has been observed to give rise to more complex dynamics in *T. cruzi*-infected *R. prolixus* [20]. We assumed that infected vectors had slightly lower fitness than uninfected vectors [20–22], represented by a scalar term *f* that could take values between 0–1.

1 $$ \raisebox{1ex}{$ dB$}\!\left/ \!\raisebox{-1ex}{$dt$}\right.=r\left(B+fV\right)\left(\frac{K-\left(B+V\right)}{K}\right)-\frac{\beta B\left({c}_a{I}_a+{c}_i{I}_i+{c}_d{I}_d+{c}_R{I}_R\right)}{R+N}-{\mu}_bB $$

2 $$ \raisebox{1ex}{$dV$}\!\left/ \!\raisebox{-1ex}{$dt$}\right.=\frac{\beta B\left({c}_a{I}_a+{c}_i{I}_i+{c}_d{I}_d+{c}_R{I}_R\right)}{R+N}-{\mu}_bV $$

We assumed that the vectors had simple dynamics driven by logistic style growth such that their abundance settles to a carrying capacity, *K* [23]. Initially we assume that K is independent of host abundance, but we also explore scenarios where vector carrying capacity is a function of the abundance of synanthropic animals that serve as an additional food source.

### Host dynamics

We represented the total human population size by N, in which there are three stages of Chagas disease: acute infections (I_a_), recently acquired and lasting 4 to 8 weeks [24]; chronic indeterminate stage infections (l_i_), a long-term infectious period with no apparent symptoms; and chronic determinate stage infections (l_d_), infectious persons that develop clinically apparent symptoms (develops in 20-30 % of those in the l_i_ stage over 10 to 30 years), and can result in death. We assumed that individuals in I_a_ are the most infectious to triatomine bugs [25, 26] and I_i_ are the least infectious [27]. Humans move into the I_a_ class through an infective contact with vectors (V) at a rate of *βc*_*vN*_, after which they move from I_a_ to l_i_ at a rate of delta (*δ*) and from l_i_ to l_d_ at a rate of sigma *(σ)*. Individuals in l_d_ have a Chagas disease related mortality rate of alpha (*α*). We assumed no superinfection of long-term patients with acute new infections. Although the time spent in each class of infection is assumed to be distributed exponentially, the net effect of allowing infections to pass through these different classes of infection is to create a more rectangular distribution of total time from initial infection to death in the final terminal infection class, as this formulation captures the main details that we are interested in. This results in three equations (3, 4, and 5) for the human population, with N-(I_a_ + I_i_ + I_d_) being the number of uninfected human hosts.

3 $$ \raisebox{1ex}{$d{I}_a$}\!\left/ \!\raisebox{-1ex}{$dt$}\right.=\frac{c_{vN}\beta V\left(N-\left({I}_a+{I}_i+{I}_d\right)\right)}{N+R}-{I}_a\left(\delta +{\mu}_N\right) $$

4 $$ \raisebox{1ex}{$d{I}_i$}\!\left/ \!\raisebox{-1ex}{$dt$}\right.=\delta {I}_a-{I}_i\left(\sigma +{\mu}_N\right) $$

5 $$ \raisebox{1ex}{$d{I}_d$}\!\left/ \!\raisebox{-1ex}{$dt$}\right.=\sigma {I}_i-{I}_d\left(\alpha +{\mu}_N\right) $$

Equation 6 represents the non-human vertebrate species that are fed upon by vectors. We divided them into viable and non-viable *T. cruzi* hosts with the addition of a scalar term, p_v_ that took values between 0–1 to represent this division; thus we ignored the relative preference of vectors for different non-human host species by including this factor. We further assumed this complex of viable reservoir species to have a common average mortality rate, μ_R_.

6 $$ \raisebox{1ex}{${dI}_R$}\!\left/ \!\raisebox{-1ex}{$dt$}\right.=\frac{c_{vR}\beta \left({p}_vR-{I}_R\right)}{\left(R+N\right)}-{I}_R{\mu}_R $$

Animals moved into the infected class I_R_ through infectious contact with vectors, V at a rate of *βc*_*vR*_, with R - I_R_ being the number of uninfected synanthropic animals.

### Vector control and impact of control on R_0_

Vector control is simulated by changes in the death rates of triatomine bugs. The addition of a death rate term, *D*, to equations 1 and 2 results in the following equations:

7 $$ \raisebox{1ex}{$ dB$}\!\left/ \!\raisebox{-1ex}{$dt$}\right.=r\left(B+fV\right)\left(\frac{K-\left(B+V\right)}{K}\right)-\frac{\beta B\left({c}_a{I}_a+{c}_i{I}_i+{c}_d{I}_d+{c}_R{I}_R\right)}{R+N}-D{\mu}_bB $$

8 $$ \raisebox{1ex}{$dV$}\!\left/ \!\raisebox{-1ex}{$dt$}\right.=\frac{\beta B\left({c}_a{I}_a+{c}_i{I}_i+{c}_d{I}_d+{c}_R{I}_R\right)}{R+N}-D{\mu}_bV $$

We used these equations to produce an expression for the relationship between additional mortality due to triatomine vector control and the reduction in vector abundance.

9 $$ \frac{B}{K}=1-\frac{D{\mu}_b}{r} $$

Triatomines are driven to extinction when Dμ_b_ equals r, but it may also be possible to break the chain of transmission at lower levels of insecticide use. The critical level of insecticide use ‘D’ that leads to increased vector mortality Dμ_b_ and ultimately interruption of *T. cruzi* transmission to humans can be found by deriving an expression for the basic reproductive rate (R_0_) of Chagas disease using the next generation method [28].

10 $$ {R}_0==\sqrt{\left(\frac{\beta {c}_{vN}V}{\left(N+R\right)\left(D{\mu}_b\right)}\right)\left(\frac{\beta {c}_R{c}_{vR}{p}_vR}{\mu_R}+\frac{N}{\delta +{\mu}_N}\right)\left[\beta {c}_a+\frac{\beta {c}_i\delta }{\left(\sigma +{\mu}_N\right)}+\frac{\beta {c}_d\delta \sigma }{\left(\sigma +{\mu}_N\right)\left(\alpha +{\mu}_N\right)}\right]} $$

We then used this equation to examine the relationship between R_0_, synanthropic animal abundance and level of insecticide use (i.e., vector mortality increase).

### Data parameters and assumptions

Parameter values came from the literature (Table 1). The parameters for triatomine bug vectors were based on the species *Rhodnius prolixus* when possible. *R. prolixus* is an epidemiologically important species in northern parts of South America [29], and has average demographic rates when compared to two other key *T. cruzi* vector species, *Triatoma infestans* and *Triatoma dimidiata*. Parameter values for non-human host species were averaged between values available in the literature for dogs, cats, opossums, and guinea pigs, which are common synanthropic animals in many Chagas-endemic areas [14, 30, 31]. We assume frequency dependent transmission, as the vectors can only feed, defecate on, or be eaten by one host at a time, and the abundance of hosts determines how frequently this occurs. We ran the model with monthly time steps for a duration of 50 years. We assumed that all populations (humans, animals and bugs) were closed, (i.e., no immigration or emigration), and we assumed the human and animal population sizes to be constant.

**Table 1:** Model parameters

Symbol	Definition	Value	Reference
r	triatomine birth rate	36.0	[52]
μ_b_	triatomine mortality rate	1.73	[52]
μ_R_	non-human host mortality rate	0.5	[27, 53]
μ_N_	general human mortality rate	0.013	[54]
β	triatomine-host contact rate	41.0	[52]
c_a_	probability of infection from I_a_-B	0.61	[25]
c_i_	probability of infection from I_i_-B	0.026	[27]
c_d_	probability of infection from I_d_-B	0.2	[55]
c_R_	probability of infection from I_R_-B	0.465	[27, 30, 56]
c_vN_	probability of infection from V-N	0.00058	[57]
c_vR_	probability of infection from V-R	0.0199	[58, 59]
δ	rate of movement from I_a_-I_i_	6.0	[24]
σ	rate of movement from I_a_-I_d_	0.03	[24]
α	I_d_ mortality rate	0.10	[24]

Rates and probabilities are per contact

### Model scenarios with variation in animal presence and vector control intensity

We modeled four scenarios: human hosts only, human and animal hosts, and vector control in the presence and absence of animals. In the first scenario, humans, (*N* = 10, which represents a household or other small, closed population), were the only *T. cruzi* hosts, and we investigated the impact of the ratio of triatomine bugs to humans through changing the triatomine carrying capacity (K). Here we also examined the impact of human population size on the infection composition of humans and the triatomine bug population. In different experiments, we set K equal to 10, 50, and 100 bugs per human. As the number of triatomines in domestic settings is highly variable [32], these K values were selected to be in line with values used in other models [33, 34], and at the same time encapsulate the variation in population size reported in empirical studies [35, 36].

The second scenario further expanded the first scenario to include the animal population. We investigated the effect of animal population size and the proportion of animals that are viable *T. cruzi * hosts on the infection composition of humans and bugs. We first investigate this scenario with triatomine abundance independent of synanthropic animal abundance. We then examine how *T. cruzi* dynamics change when triatomine abundance is dependent on animal abundance by making the triatomine bug carrying capacity a linear function of synanthropic species abundance.

In the third and fourth scenario we introduced vector control into scenarios one and two, and we explored the effects of targeting triatomine carrying capacity and death rates on the infected human and bug populations. Here we used R_0_ to provide insight into the rates at which vectors need to be controlled in order to break the chain of transmission.

## Results

### Scenario 1: human hosts only

**Fig. 1:**
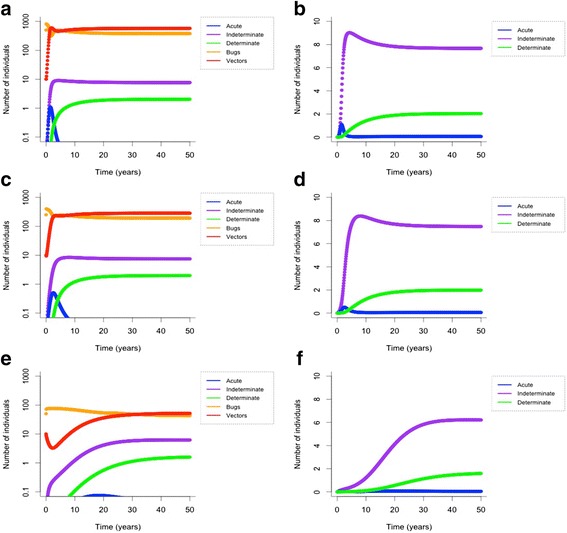
Number of *T.* cruzi-infected humans (*N* = 10) and triatomines at different carrying capacities with no animals in the transmission scenario. Top row: **a**). K = 1000, bugs and humans; **b**). K = 1000, just humans. Middle row: **c**). K = 500, bugs and humans; **d**). K = 500, just humans. Bottom row: **e**). K = 100, bugs and humans; **f**). K = 100, just humans. Starting conditions: B = K/2, V = 10, no infected humans

When holding all else constant, with humans (*N* = 10) as the only *T. cruzi* hosts, increasing the carrying capacity of triatomine bugs (i.e., the ratio of bugs to human hosts), increases the speed of *T cruzi* transmission in the system (Fig. 1). At 100 bugs per person, all 10 humans are infected with *T. cruzi* after 8.3 years. At 50 bugs per person, all 10 humans are infected with *T. cruzi* after 11.7 years. These high levels of prevalence are due to the model assumption of a closed human population. As we are mainly concerned with the interaction of vector control and synanthropic host abundance on control, we are essentially using prevalence as an index of relative risk of human infection. Prevalence begins to decrease at 10 bugs per person, where fewer than 90 % of humans have Chagas disease after 50 years, although the infections have not leveled off. Additionally, the proportion of infected bugs in the vector population remains consistent across different carrying capacities, with approximately 61 % of bugs infected at K = 1000 and K = 500, and 58 % infected at K = 100 (Fig. 1).

### Scenario 2: human and animal hosts

**Fig. 2:**
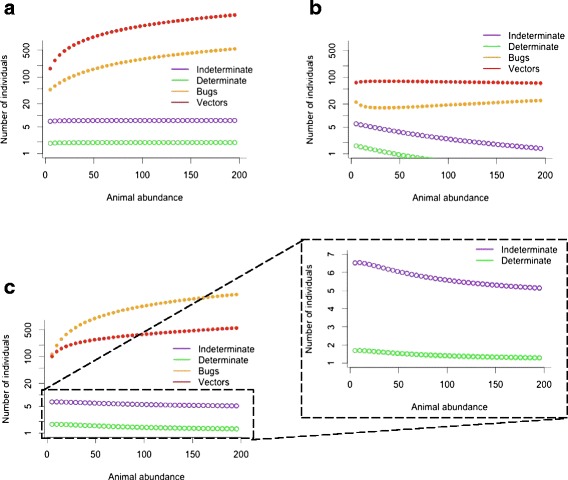
Number of *T. cruzi*-infected humans (*N* = 10) and triatomines by animal abundance. Top row: **a**). K increases linearly with animal abundance and 75 % of animals are viable *T. cruzi* hosts; **b**). K = 100 and 75 % of animals are viable hosts. Bottom row: **c**). K increases linearly with animal abundance and 1 % of animals are viable hosts. Simulated for 50 years and output from the final five years shown

The addition of synanthropic animals to the system reduces *T. cruzi* transmission speed and human infection prevalence if triatomine carrying capacity is not increased. As animal abundance rises, the proportion of the human population infected with *T. cruzi* decreases (Fig. 2b). The addition of 20 synanthropic animals (75 % viable *T. cruzi* hosts) reduces the speed of *T. cruzi* transmission to humans (*N* = 10), compared to when there are only human hosts. At K = 1000, all 10 humans are infected with *T. cruzi* after 9.3 years, one year later than without animals (8.3 years). At 50 bugs per person (K = 500), all 10 humans are infected with *T. cruzi* after 15.3 years (compared to 11.7 years without animal hosts). At 10 bugs per person (K = 100), there are still fewer than 8 people with Chagas disease after 50 years (one person fewer than without animals), although the number of human infections slowly continues to increase. Additionally, transmission speed is further reduced as the proportion of animals that are viable *T. cruzi* hosts decreases.

Although the addition of synanthropic animals (*n* = 20) slows *T. cruzi* transmission to humans, it also increases the proportion of the triatomine bug population infected with *T. cruzi*. For K values of 1000 and 500, the population stabilizes at about 85 % of the bugs infected (75 % of animals viable), as opposed to 61 % infected without animal hosts. At K = 100, 83 % of bugs are infected, up from 58 % without animals. This effect is reduced as animal* T. cruzi* host competence decreases.

With triatomine bug abundance dependent on the number of the synanthropic animals in the system, *T. cruzi* transmission speed increases dramatically. With carrying capacity increased by 25 bugs for each animal introduced into the system and 75 % of animals viable *T. cruzi* hosts, the number of humans infected with *T. cruzi* increases slowly at all animal abundances between 5 and 200 (Fig. 2a). When most of the animals in the system are not viable *T. cruzi* hosts, *T. cruzi* transmission is slower, and the number of humans with Chagas disease begins to decline slowly as animal abundance increases (Fig. 2c and d).

### Scenario 3: triatomine bug control, no animals present

The speed of *T. cruzi* transmission to humans (*N* = 10, K = 1000) is reduced with control strategies that increase the triatomine death rate by ≥50 % from the background vector mortality rate. For example, with a 75 % increase in vector death rate, the human population saturates with *T. cruzi* infection after 9 years, compared to 8.3 years with no intervention. Doubling the triatomine death rate, slows *T. cruzi* transmission even more, with saturation occurring after 11.8 years. While these increased death rates reduce the speed of *T. cruzi* transmission in the human population, all humans still eventually become infected. The number of humans with Chagas disease is only reduced after the triatomine bug death rate is increased by at least 7.

At a triatomine carrying capacity of 500, the dynamics are more sensitive to increases in triatomine death rate, with a 25 % death rate increase slowing saturation to 14.3 years. The number of humans with Chagas disease begins to decrease when triatomine mortality is increased by 3.25 times. At K = 100 and a 25 % increase in triatomine mortality, there are fewer than 8 people with Chagas disease after 50 years, compared with 9 people with no vector control.

### Scenario 4: triatomine bug control with animals present

The addition of 20 animals (75 % viable, *N* = 10) to the system makes the dynamics more sensitive to changes in triatomine death rates. *T. cruzi* transmission to humans is slower at vector death rate increases of 1 % and higher. For example, with a vector death rate increase of 25 %, saturation of humans infected with Chagas disease occurred after 9.7 years when K = 1000 (compared to 9 years with no animals and intervention), and 15.9 years (compared to 14.3 years when K = 500). However, there is still no change in the final number of humans with Chagas disease until triatomine the death rate was increased 7-fold when K = 1000 and 3.5 fold for K = 500.

### R_0_ and vector control in the presence of animals

**Fig. 3:**
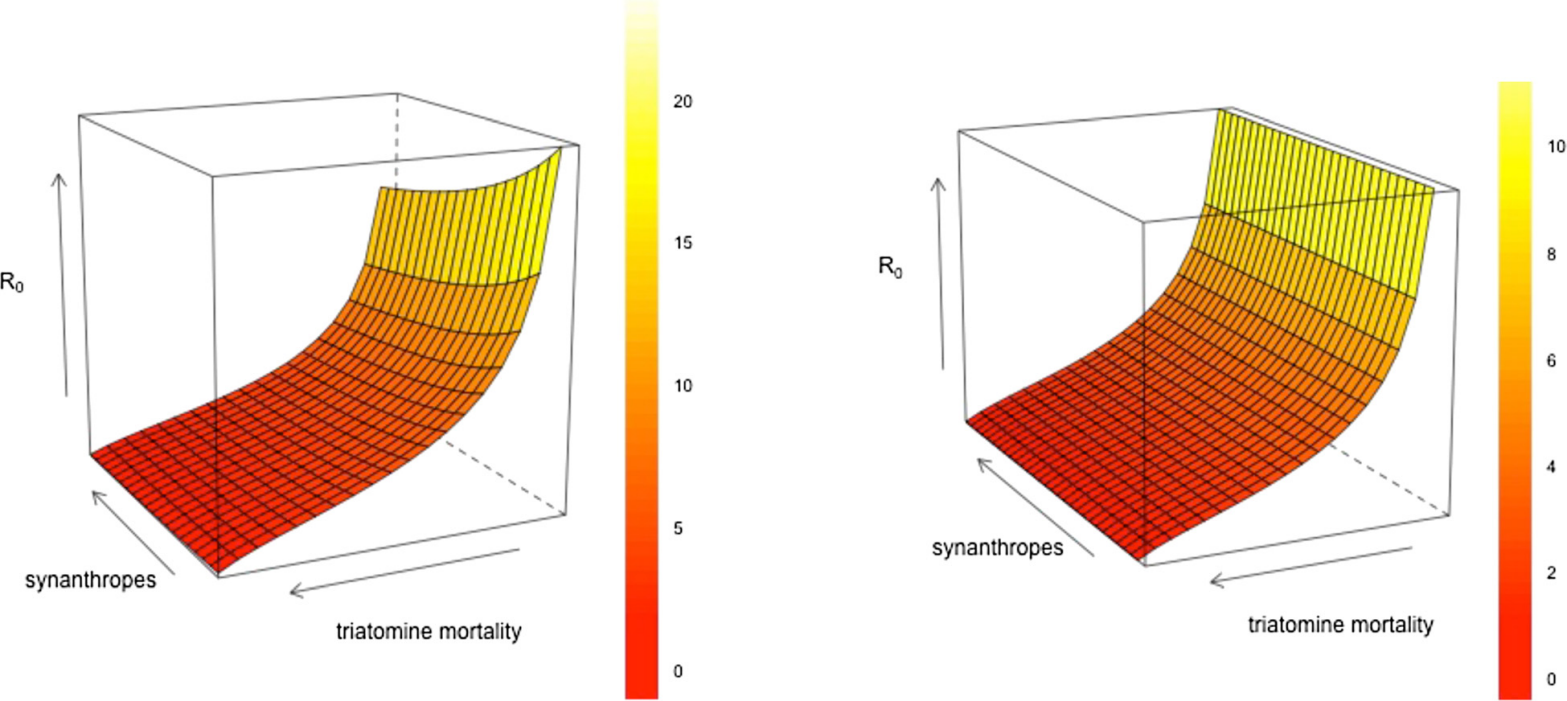
The relationship between R_0_, synanthropic animal abundance and triatomine mortality. Top: System with 10 humans. Bottom: System without humans. Run with 500 infected bugs (i.e., 'vectors') and 10 human hosts for triatomine mortality increases between 1–20 times the background rate, and 1–30 synthropic animals, of which 75 % are competent *T. cruzi* hosts

R_0_decreases as both synanthropic animal abundance and vector mortality increase (Fig. 3). With 75 % of animals viable *T. cruzi* hosts and triatomine mortality doubled (*N* = 10, V = 500), R_0_ ranges from 21.42 (2 animals) to 16.14 (20 animals). With 25 % viable hosts and mortality doubled, R_0_ ranges from 21.21 (2 animals) to 14.01 (20 animals). R_0_ falls below one when the triatomine mortality rate is increased by a factor of 20 and there are at least two animals.

Without humans, R_0_ is further reduced (Fig. 3, bottom image). With a doubled vector mortality rate R_0_ is 12.04 (75 % viable animal hosts) and 6.95 (25 % viable) at all animal population sizes between 2–30. R_0_ drops below one when vector mortality is increased at least 16 times when 25 % of the animals are viable, and 19 times when 75 % of animals are viable.

## Discussion

Our results suggests that vector control methods targeting triatomine death rates will reduce the speed of *T. cruzi* transmission to humans, but must be implemented at very high intensities to reduce Chagas disease prevalence and R_0_. The addition of synanthropic animals decreases the speed of *T. cruzi* transmission to humans if these hosts have no effect on triatomine abundance. However, when synanthropic animals increase triatomine carrying capacity, then higher levels of vector control are required to reduce transmission, particularly if the animals are competent *T. cruzi* hosts.

### Synanthropic animals: a dilution effect?

Although it is not unexpected that the addition of 20 animals into the system slowed *T. cruzi* transmission to humans, as it diluted the ratio of bugs to hosts by two-thirds, the viable animal hosts in our model did have a higher probability of becoming infected themselves and also of infecting the triatomine bug, which is reflected in the higher proportion of infected bugs when they are added to the system. Therefore, the possibility remained that they could amplify transmission as well, even without increasing triatomine carrying capacity. Surprisingly, our simulation of R_0_ revealed that, at comparable carrying capacities, it is the humans that amplify transmission, probably because of their long life spans.

The ‘dilution effect’ hypothesis is defined as a decrease in infectious disease risk with an increase in species diversity [37, 38]. Although in our model we do not delineate between species beyond human and non-human, our results suggest that zooprophylaxis could occur with the addition of non-human hosts that divert *T. cruzi-*infected triatomine bites away from humans. Moreover, *T. cruzi* is considered as a parasite that responds negatively to biodiversity in undisturbed ‘wild’ systems [39], although it is unknown if this is the case in domestic transmission settings. However, arguable dilution effects have been observed in peri-domestic transmission scenarios around the Panama Canal [40]. Thus, our results support the possibility of a dilution effect, but future development of the model to include different animal species is needed and will be explored in future work.

### The carrying capacity crux

Our results suggest that if measures are taken to prevent triatomine abundance from increasing with the addition of synanthropic animals, then they would not only be beneficial, but it could be possible to keep the *T. cruzi* R_0_ below 1, even without driving the triatomine population to zero. However, this is not an easy task, as synanthropic animals in domestic and peri-domestic transmission scenarios lead to an increased blood (i.e., food) supply for triatomine bugs. As obligate blood feeders, the number of eggs laid by a female triatomine is strongly correlated with the amount of blood consumed [41], so an increase in blood availability generally leads to an increased carrying capacity if sufficient triatomine habitat is available, (illustrated in the iteration of our model with triatomine density dependent on animal abundance). Therefore, triatomine carrying capacity must be reduced, or at least prevented from increasing in the presence of synanthropic animals. This is currently done with varying levels of success through housing improvements that include replacing roofing and wall materials [42], to reduce the dark and hidden microspaces preferred by domiciliary triatomines [43]. Another potential area of housing improvement is targeting abiotic factors in triatomine microhabitats such as climate [44], light and substrate [45], factors to which triatomine bugs are very sensitive.

### Policy implications and the 2020 goals

Our results have several policy implications. First, as stated above, to impact the magnitude of *T. cruzi* spread, prevention and control measures must focus on decreasing triatomine abundance in domestic settings. After the triatomine bugs reach a certain carrying capacity, only the speed of *T. cruzi* transmission will be impacted by interventions that fall short of severely reducing the population and preventing its subsequent re-infestation.

Our results suggest that the two strategies (IRS and housing improvements) for meeting the 2020 goal of interrupting domestic vector-borne transmission, could theoretically achieve this goal. This would require a centralized and sustained campaign to employ these methods in a large enough number of triatomine-infested dwellings across all 21 countries with vector-borne *T. cruzi* transmission, which may be extremely difficult to achieve, as there are a number of challenges in performing such an operation. These challenges include the lack of a centralized agency with the willingness and the resources to organize such an extensive campaign against Chagas disease. This in turn is further complicated by the decentralization of vector-borne disease control programs in many countries [46, 47]; the existence of many Chagas-endemic areas located in armed conflict zones (O. Cantillo and M. Vera, pers. communication [Colombia]); and competition for funding with other better-known vector-borne diseases, such as dengue fever and Chikunguyna [46, 48]. Moreover, Chagas disease patterns are highly heterogeneous, even within the same country. With a lack of sufficient baseline prevalence and/or little to no data for many areas, designing a vector control campaign with a far enough reach to eliminate Chagas disease transmission by 2020 (i.e., within the next three years) seems extremely difficult. Given these obstacles, other prevention and control measures should continue to be considered and developed for Chagas disease (e.g., early diagnosis, new medications and vaccines, etc.). Studies have shown that a Chagas vaccine could be cost-effective and could even garner a positive return-on-investment fairly early after its introduction [49, 50].

### Future developments

All models are simplifications of real-life and therefore cannot account for every possible event or outcome [51]. Our results are intended to be broad, and do not account for the enormous amount of variability found in every aspect of *T. cruzi* transmission, including variation in parasite strain, the health status of each individual host upon infection, variation in triatomine species efficiency as *T. cruzi* vectors, and variation in the level and duration of *T cruzi* parasitemias found across different mammal species. Additionally,diversity in *T. cruzi* competence between animal species no doubt adds complexity to Chagas disease dynamics. For instance, individual animals that sustain a high parasitemia for relatively long periods of time can amplify *T. cruzi,* as has been observed in Peruvian guinea pigs [14]. Moreover, the incorporation of triatomine host preference will shift the dynamics of the system and in some scenarios could lower the human-triatomine contact rate, which has been predicted to occur under some circumstances in the presence of dogs and chickens [11]. There will also be environmental variability due to geographic location, in addition to cultural diversity that will influence human behavior. All of these are important factors to keep in mind for future models of *T. cruzi* transmission.

## Conclusion

Our results suggest that in domestic *T. cruzi* transmission scenarios where no vector control measures are in place, a reduction in synanthropic animals may slow *T. cruzi* transmission to humans, but it would not lead to the complete interruption of transmission. We found that it is more critical to target vector abundance than synanthropic animals, and, in scenarios where measures are taken to control triatomine population growth, synanthropic animals could play a beneficial role by decreasing the speed of *T. cruzi* transmission to humans, and increasing the sensitivity of the system to vector control measures. More work is needed to quantify the extent of this effect in different transmission scenarios, and we do not recommend adding synanthropic animals to any system before this is studied further. Therefore, to reach the 2020 goal of interrupting intra-domiciliary *T. cruzi* transmission, control measures must continue to aggressively target domestic vector populations.

## Abbreviations

*T. cruzi*: *Trypanosoma cruzi*
WHO: World Health Organization
*R. prolixus*: *Rhodnius prolixus*
IRS: Indoor residual spraying
